# Extracellular vesicles mediate inflammasome signaling in the brain and heart of Alzheimer’s disease mice

**DOI:** 10.3389/fnmol.2024.1369781

**Published:** 2024-04-10

**Authors:** Brianna Cyr, Erika D. L. R. M. Cabrera Ranaldi, Roey Hadad, W. Dalton Dietrich, Robert W. Keane, Juan Pablo de Rivero Vaccari

**Affiliations:** ^1^Department of Neurological Surgery and The Miami Project to Cure Paralysis, University of Miami Miller School of Medicine, Miami, FL, United States; ^2^Department of Physiology and Biophysics, University of Miami Miller School of Medicine, Miami, FL, United States

**Keywords:** inflammasome, heart, caspase-1, ASC, inflammation, Alzheimer’s Disease, Extracellular Vesicles

## Abstract

**Introduction:**

Alzheimer’s disease (AD) is an inflammatory neurodegenerative disease characterized by memory loss and cognitive impairment that worsens over time. AD is associated with many comorbidities, including cardiovascular disease that are associated with poorer outcomes. Comorbidities, especially heart disease and stroke, play a significant role in the demise of AD patients. Thus, it is important to understand how comorbidities are linked to AD. We have previously shown that extracellular vesicle (EV)-mediated inflammasome signaling plays an important role in the pathogenesis of brain injury and acute lung injury after traumatic brain injury.

**Methods:**

We analyzed the cortical, hippocampal, ventricular, and atrial protein lysates from APP/PS1 mice and their respective controls for inflammasome signaling activation. Additionally, we analyzed serum-derived EV for size, concentration, and content of inflammasome proteins as well as the EV marker CD63. Finally, we performed conditioned media experiments of EV from AD patients and healthy age-matched controls delivered to cardiovascular cells in culture to assess EV-induced inflammation.

**Results:**

We show a significant increase in Pyrin, NLRP1, caspase-1, and ASC in the brain cortex whereas caspase-8, ASC, and IL-1β were significantly elevated in the heart ventricles of AD mice when compared to controls. We did not find significant differences in the size or concentration of EV between groups, but there was a significant increase of caspase-1 and IL-1β in EV from AD mice compared to controls. In addition, conditioned media experiments of serum-derived EV from AD patients and age-matched controls delivered to cardiovascular cells in culture resulted in inflammasome activation, and significant increases in TNF-α and IL-2.

**Conclusion:**

These results indicate that EV-mediated inflammasome signaling in the heart may play a role in the development of cardiovascular diseases in AD patients.

## 1 Introduction

Alzheimer’s disease (AD) is a progressive neurodegenerative disorder affecting an estimated 6.7 million people in the United States alone (Alzheimer’s Association Report, 2023). AD is characterized by cognitive and memory decline that worsens over time due to the accumulation of amyloid-β (Aβ) plaques and neurofibrillary tangles (NFT). AD pathogenesis is associated with central nervous system (CNS) inflammatory responses, oxidative stress, and neuronal death.

A key component of the innate inflammatory response is the inflammasome. The inflammasome leads to the production of interleukin (IL)-1β and IL-18 via activation of caspase-1 (De Rivero Vaccari et al., 2016b). The inflammasome is a multi-protein complex comprised of a sensor protein such as a NOD-like receptor (NLR) protein, apoptosis-associated speck-like protein containing a caspase recruitment domain (ASC), and pro-caspase-1 (Martinon et al., 2002). We have previously shown that the inflammasome is a significant contributor to the inflammatory response in the CNS (De Rivero Vaccari et al., 2016b) and that CNS injury results in a systemic inflammatory response that extends to other tissues by a mechanism that is mediated in part by extracellular vesicles (EV) (Kerr et al., 2020). In addition, injury to the brain results in the release of EV that triggers inflammation in the heart of mice as part of a neural-cardiac inflammasome axis (Keane et al., 2023).

In AD, an abnormal accumulation of Aβ is cleared by microglia, the resident immune cells of the brain (Lee and Landreth, 2010). Microglia express triggering receptor expressed on myeloid cells 2 (TREM2) which aids in Aβ clearance by binding Aβ, promoting microglial phagocytosis and survival, as well as by modulating inflammation (Hou et al., 2022). AD patients exhibit activation of the NLRP1 and NLRP3 inflammasomes in monocytes with significantly higher amounts of IL-1β and IL-18 compared to controls (Saresella et al., 2016). Moreover, we have previously shown that ASC is significantly elevated in the serum of mild cognitively impaired (MCI) patients when compared to controls and AD patients, suggesting that ASC plays an important role in the early stages of AD (Scott et al., 2020). In addition, traumatic brain injury exacerbates inflammation in the brain by a mechanism that is, in part, mediated by the inflammasome (Johnson et al., 2023a,b).

EV, including microvesicles, exosomes, and apoptotic bodies, are membrane-bound vesicles secreted by cells into bodily fluids including blood, CSF, urine, and respiratory secretions. EV play a role in pro-inflammatory and anti-inflammatory conditions, depending on their cargo. There is increasing evidence that EV play a role in the maintenance of normal physiological conditions such as tissue repair, immune surveillance, and blood coagulation, as well as in the pathology of several diseases (Shetty and Upadhya, 2021). Moreover, IL-18 is released as a product of inflammasome activation and is associated with EV that are shed from the surface of macrophages. In addition, Aβ is secreted from EV (Rajendran et al., 2006) and EV-associated Aβ levels are significantly increased in APP transgenic mice (Yuyama et al., 2015), suggesting that EV enhance Aβ aggregation and plaque formation (Dinkins et al., 2014). Lastly, total tau levels in EV are higher in AD patients than in controls (Fiandaca et al., 2015).

Comorbidities such as cardiovascular disease and bronchopneumonia are more significant in AD patients than in age-matched controls and are also associated with poorer outcomes in AD (Zhao et al., 2008; Duthie et al., 2011). AD pathology is associated with genetic factors such as the apolipoprotein E4 (ApoE4) allele and variants in presenilin 1 and 2 (PSEN1, PSEN2). These genes have also been associated with cardiomyopathy (Li et al., 2006; Gianni et al., 2010). Moreover, heart failure results in cerebral hypoperfusion likely due to a decrease in systolic function (Tini et al., 2020). In addition, cerebral hypoperfusion after atrial fibrillation contributes to deposition of Aβ plaques and NFTs in the brain (Dublin et al., 2014). Furthermore, Aβ has been found to be present in the heart of AD patients (Troncone et al., 2016). However, the role of EV in cardiac dysfunction in AD patients remains unclear.

In this study, we investigated inflammasome signaling protein levels in the brain and heart of APP/PS1 and WT control mice. Additionally, we examined size, concentration, and contents of EV from the serum of APP/PS1 and WT control mice and conducted adoptive transfer experiments of EV from AD patients and age-matched controls into cardiovascular cells to determine the effects of EV containing a cargo of inflammasome proteins on the inflammatory response in the cardiovascular system.

## 2 Materials and methods

### 2.1 Animals

All animal procedures were approved by the Animal Care and Use Committee of the University of Miami (protocol 19–164). Animal procedures were carried out according to the Guide for the Care and Use of Laboratory Animals (U.S. Public Health). Male B6; C3-g (APPswe, PSEN1dE9)85Dbo/Mmjax (APP/PS1) mice (Jackson Laboratories/MMRRC, MMRRC Strain #034829-JAX) (Jankowsky et al., 2001, 2004; Reiserer et al., 2007) and their respective non-carrier controls were used. Animals were sacrificed at 6 months of age, the cerebral cortex and heart of each animal were then removed, and protein lysates were obtained; each brain was dissected into cortex and hippocampus and each heart was dissected into atria and ventricles. Lysed protein samples were then stored at -80°C until analyses. Blood was collected by cardiac puncture and allowed to clot at room temperature followed by centrifugation at 2,000 rpm for 10 min in a refrigerated centrifuge. The resulting supernatant was then designated as serum. Following centrifugation serum samples were stored at -80°C until processed for EV analyses.

### 2.2 Immunoblotting

Brain and heart protein lysates were obtained as described in (Mejias et al., 2018), and lysates were then resolved by immunoblotting for the expression of inflammasome signaling proteins as in (Cyr and de Rivero Vaccari, 2023a). Briefly, lysates were resolved in 4–20% Criterion TGX Stain-Free precast gels (Bio-Rad), using antibodies at a dilution of 1: 1,000. Primary antibodies used in this study were against the following proteins: NLRP1 (Novus Biologicals), NLRP3 (Novus Biologicals), AIM2 (eBioscience), Pyrin (Santa Cruz), caspase-1 (Novus Biologicals), ASC (Santa Cruz), IL-1β (Cell Signaling), caspase-8 (Novus Biologicals), CD63 (Novus Biologicals), and β-actin (Sigma Aldric). Quantification of band densities was done using the UN-SCAN-IT gel 6.3 Software (Silk Scientific Corporation). Membranes were imaged using the ChemiDoc Touch Imaging System (BioRad) following chemiluminescence.

### 2.3 Partial purification of ASC specks

ASC specks were partially purified as previously described (Adamczak et al., 2014). Briefly, heart lysates were filtered with 5 μm polyvinylidene difluoride membrane (Millipore) at 2,000 xg for 5 min. The filtered supernatant was centrifuged at 5,000 rpm for 8 min and the pellet was resuspended in CHAPS buffer. The pyroptosome was pelleted by centrifugation at 5,000 rpm for 8 min. The pellet was resuspended in CHAPS buffer and disuccinimidyl suberate (DSS) for 30 min at room temperature to cross-link ASC dimers. An equal volume of 2x Laemmli was added and samples were immunoblotted for ASC as described above.

### 2.4 Blood pressure, oximetry, and heart rate

Before the mice were sacrificed, blood pressure, oximetry, and heart rate were recorded. Blood pressure was measured using the CODA monitor (Kent Scientific). The tails of mice were inserted into the tail cuff and blood pressure was taken. Heart rate and oximetry were measured using the MouseSTAT® Jr. (Kent Scientific). Readings were taken from the paw of the mice.

### 2.5 Extracellular vesicle isolation

EV were isolated with magnetic beads using the Exosome Isolation kit for mouse (Miltenyi Biotec) according to manufacturer’s instructions. Briefly, 100 μL of serum were magnetically labeled with isolation microbeads for 1 h. Then μ columns were placed in the magnetic field of the μMACS separator attached to a MACS MultiStand and equilibrated with 100 μL of equilibration buffer, followed by rinsing with isolation buffer. The magnetically labeled samples were then added to the μ columns followed by washing steps and elution of EV by removing the μ columns from the magnetic field and adding 100 μL of isolation buffer to each μ column and immediately flushing the EV by pushing a plunger into the μ column.

### 2.6 Nanoparticle tracking analysis

Isolated EV were analyzed for particle size and particle concentration with the NanoSight NS300 instrument (Malvern Instruments Company, Nanosight, and Malvern) using Nanosight NTA 2.3 software as in (Kerr N. et al., 2018; Kerr N. A. et al., 2018; Kerr et al., 2019; Raval et al., 2019). Briefly, 2 μL of EV were added to 998 μL of distilled (DI) water to prepare the NTA sample. The instrument was first flushed with approximately 3 mL of DI water. Approximately 300 μL of sample were loaded into the NanoSight NS300 for analysis.

### 2.7 Conditioned media experiments

For conditioned media experiments, EV were isolated from the serum of AD patients and healthy age-matched controls (IRB#20170439) using Total Exosome Isolation Reagent (Invitrogen). Serum from 6 AD patients (47 to 79 years old, 5 males and 1 female) and 6 healthy controls (56 to 67 years old, 4 females and 2 males) was purchased from BioIVT as described in (Scott et al., 2020). Subsequently, 100 uL of serum were incubated with 20uL of Total Exosome Isolation Reagent (from serum) for 30 min at 4^o^ C. After incubation, samples were centrifuged at 10,000 xg at room temperature for 10 min. At the end of centrifugation, the supernatant was aspirated, and the EV pellet was resuspended in 1 mL of Smooth Muscle Cell Growth Medium (Cell Applications).

Adoptive transfer of EV from AD and healthy controls was completed in T/G HA-VSMC human cardiovascular cells (American Type Culture Collection (ATCC)). Cells were cultured with Smooth Muscle Cell Growth Medium (Cell Applications, San Diego, CA, United States) supplemented with 10% fetal bovine serum (FBS) (GeminiBio). Cells were grown in a T-75 flask until 70% confluency, after which they were transferred to a 24-well plate (~4.3×10^7^ particles/mL) as in (Keane et al., 2023). Media was aspirated and replaced by 1 mL of isolated EV resuspended in Smooth Muscle Cell Growth Medium; cells were then placed in an incubator at 37^o^ C for 2 h and the cell medium was harvested after termination of EV exposure. Caspase activity in the cell medium was assessed as per manufacturer instructions using the Caspase-Glo® 1 Inflammasome Assay (Promega, Madison, WI, United States). Luminometry was quantified in the SPARK 10 M (TECAN) spectrophotometer.

### 2.8 Electrochemiluminescence immunoassay

The inflammatory cytokines IL-2 and tumor necrosis factor (TNF)-α were measured following adoptive transfer of EV from AD patients and healthy controls into T/G HA-VSMC human cardiovascular cells (American Type Culture Collection (ATCC)) using V-Plex technology according to manufacturer instructions (MSD) using the MESO-QuickPlex SQ-120 (MSD) as previously described in (Scott et al., 2020, 2022).

### 2.9 Statistical analyses

Prism 9.0 software (GraphPad Software) was used for statistical analyses. Normality was determined by the Shapiro–Wilk test. Statistical comparisons between two groups were done using a student’s t-test for parametric data or a Mann–Whitney test for non-parametric data. *p*-values of significance used were < 0.05. Outcome measures were evaluated by investigators who were blinded to experimental groups.

## 3 Results

### 3.1 Inflammasome signaling proteins are elevated in the cortex of AD mice

**Figure 1:**
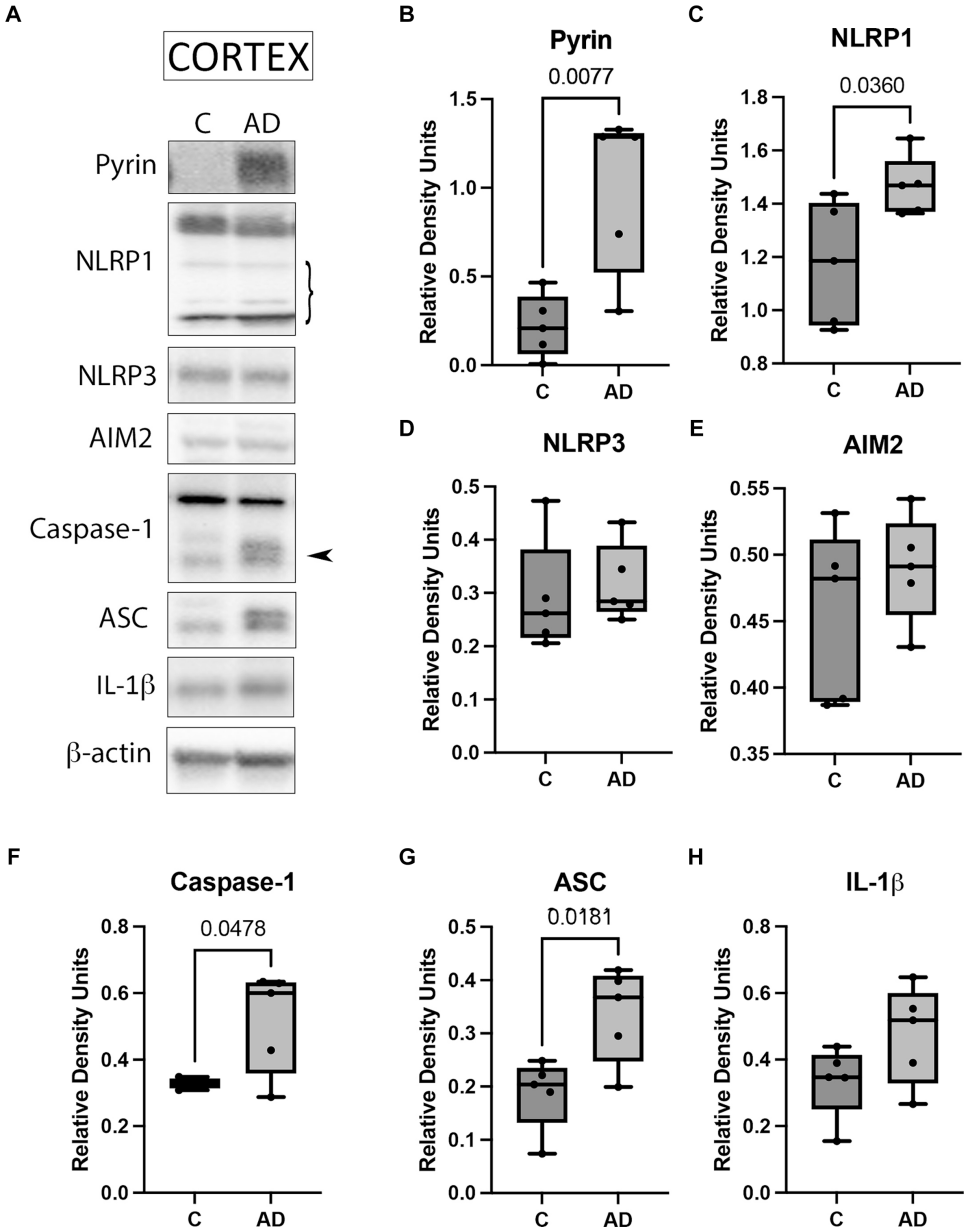
Inflammasome activation in the cortex of APP/PS1 mice. **(A)** Representative immunoblot of inflammasome proteins in the cortex of WT control (C) and APP/PS1 (AD) mice. Protein expression of **(B)** pyrin, **(C)** NLRP1, **(D)** NLRP3, **(E)** AIM2, **(F)** caspase-1 arrow points at the active form of caspase-1 (quantified), **(G)** ASC, and **(H)** IL-1β. Data were normalized to β-actin. Data presented as box plots with the min to max showing all points. *N*: 5 per group.

Since the inflammasome contributes to the pathology of AD (Vontell et al., 2023), we aimed to determine protein levels of the key inflammasome signaling components to establish which inflammasomes contribute to AD in the cortex of APP/PS1 mice. Cortical protein lysates of WT and APP/PS1 mice were immunoblotted for inflammasome protein expression (Figure 1A). The protein levels of Pyrin (Figure 1B), NLRP1 (Figure 1C), caspase-1 (Figure 1F), and ASC (Figure 1G) were significantly elevated in AD mice when compared to control. However, we did not detect a significant difference in the protein levels of NLRP3 (Figure 1D), AIM2 (Figure 1E), and IL-1β (Figure 1H). However, the latter showed a trend of higher levels in APP/PS1 mice when compared to WT mice. In addition, since AD is also associated with loss of neurons in the hippocampus (Vontell et al., 2023), we determined by immunoblotting the protein levels of the inflammasome components in hippocampal protein lysates of WT and APP/PS1 mice (Supplementary Figure S1A). However, none of the proteins analyzed (Supplementary Figures S1B–H) differ in APP/PS1 mice when compared to WT. Together, these results indicate that there is activation of the Pyrin and NLRP1 inflammasomes in the cortex of APP/PS1 mice.

### 3.2 Inflammasome signaling proteins are elevated in the heart of AD mice

**Figure 2:**
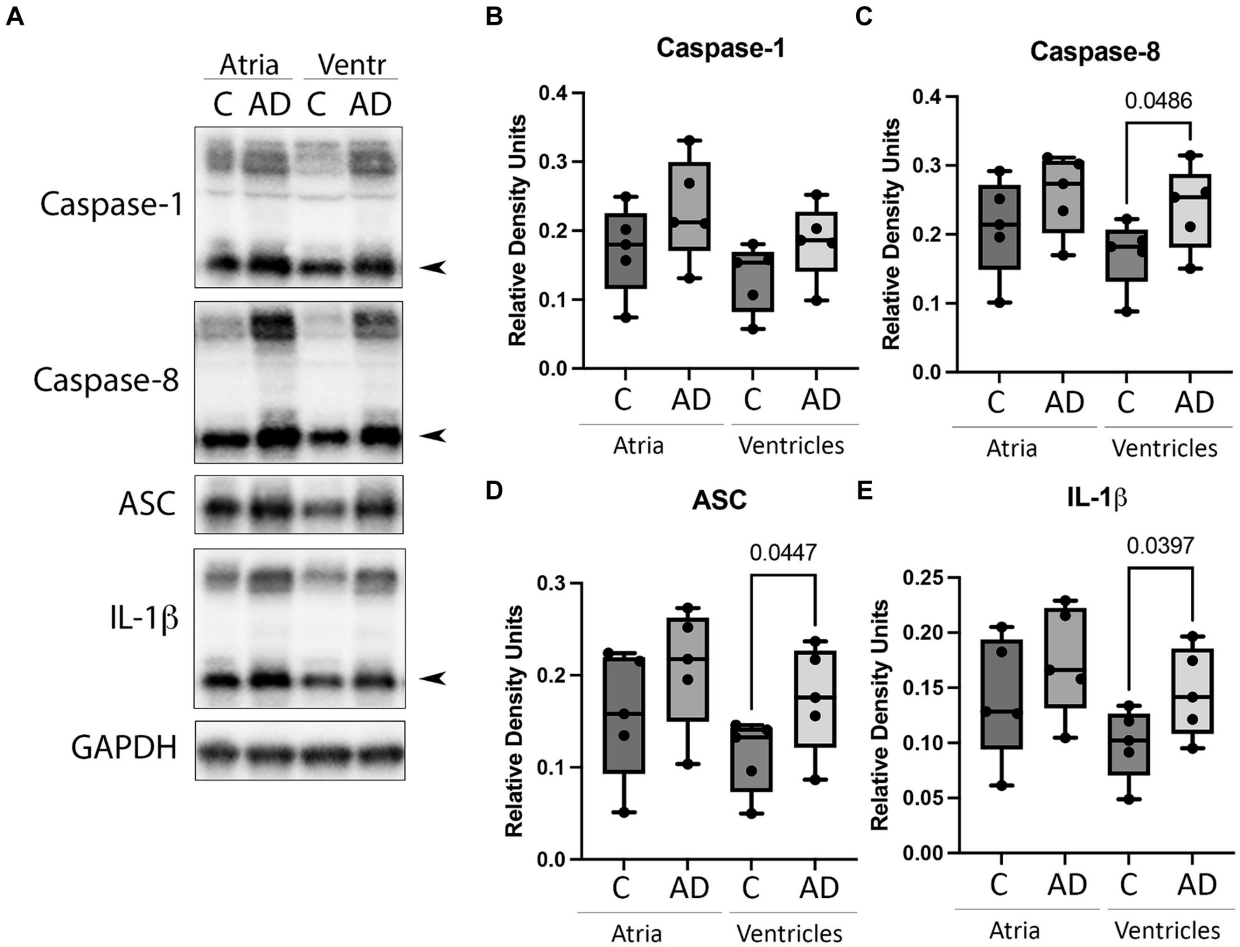
Inflammasome protein expression in the heart of AD mice. **(A)** Immunoblot of the atria and ventricles of APP/PS1 (AD) and WT control (C) mice blotted for **(B)** Caspase-1, **(C)** Caspase-8, **(D)** ASC, and **(E)** IL-1β. Data presented as box plots with the min to max showing all points. *N* = 5 per group. Quantified caspase-1, caspase-8, and IL-1β corresponds to the active (cleaved) form (arrow).

Cardiovascular comorbidities are common among AD patients, and inflammation in the heart can lead to cardiac diseases (Dick and Epelman, 2016). To determine if the inflammasome is activated in the heart in AD, we determined by immunoblotting the protein levels of canonical and non-canonical inflammasome signaling proteins. Atrial and ventricular lysates of WT and APP/PS1 mice were immunoblotted for inflammasome protein expression (Figure 2A). Quantification of band densities indicated a significant increase in the ventricles of APP/PS1 mice for caspase-8 (Figure 2C), ASC (Figure 2D), and IL-1β (Figure 2E) when compared to WT. However, there was not a significant difference in the levels of caspase-8, ASC, and IL-1β in the atria or in caspase-1 (Figure 2B) in both the atria and ventricles. These results indicate that in AD, inflammasome proteins are activated in the heart in addition to the cerebral cortex.

### 3.3 ASC speck formation in the heart of AD mice

**Figure 3:**
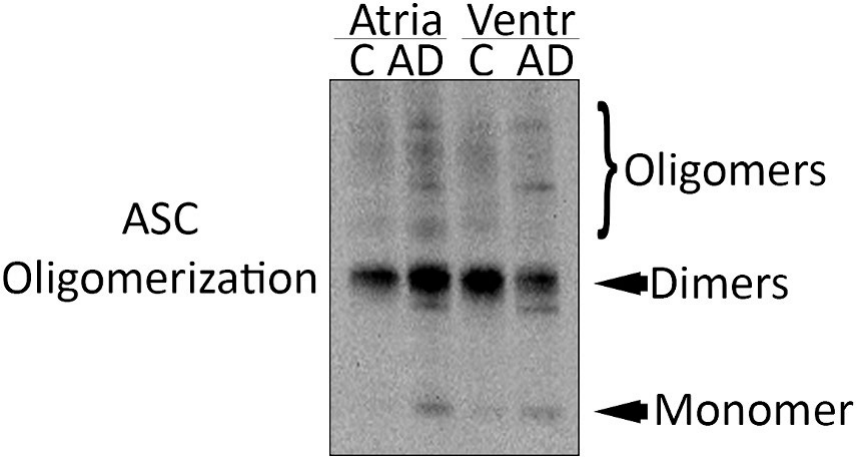
ASC speck formation in the heart of AD mice. Representative immunoblot of the partial pyroptosome purification of the atria and ventricles of WT control (C) and APP/PS1 mice (AD) immunoblotted for ASC.

ASC oligomerizes in a prion-like fashion forming ASC specks, and inflammasome activation leads to the formation and release of ASC specks (Franklin et al., 2014). ASC specks cross-link with Aβ, suggesting a role in AD (Venegas et al., 2017). To determine if there is ASC speck formation in the hearts of AD mice, we isolated the pyroptosome from atrial and ventricular lysates and immunoblotted them for ASC expression (Figure 3). Our data show that there is an increase in ASC oligomerization in the atria and ventricles of AD mice compared to WT, suggesting that ASC speck formation increases in the heart of APP/PS1 mice.

### 3.4 Blood pressure, heart rate, and oximetry of AD mice

Cardiovascular dysfunction and AD are linked (Li et al., 2006; Gianni et al., 2010; Troncone et al., 2016; Tini et al., 2020). To determine cardiovascular function, we measured the systolic blood pressure (Supplementary Figure S2A), diastolic blood pressure (Supplementary Figure S2B), mean blood pressure (Supplementary Figure S2C), heart rate (Supplementary Figure S2D), and oximetry (Supplementary Figure S2E) of WT and APP/PS1 mice. Although not significant, we found that on average, AD mice present lower blood pressure and oxygen saturation than age-matched controls.

### 3.5 Serum-derived EV characterization in AD mice

**Figure 4:**
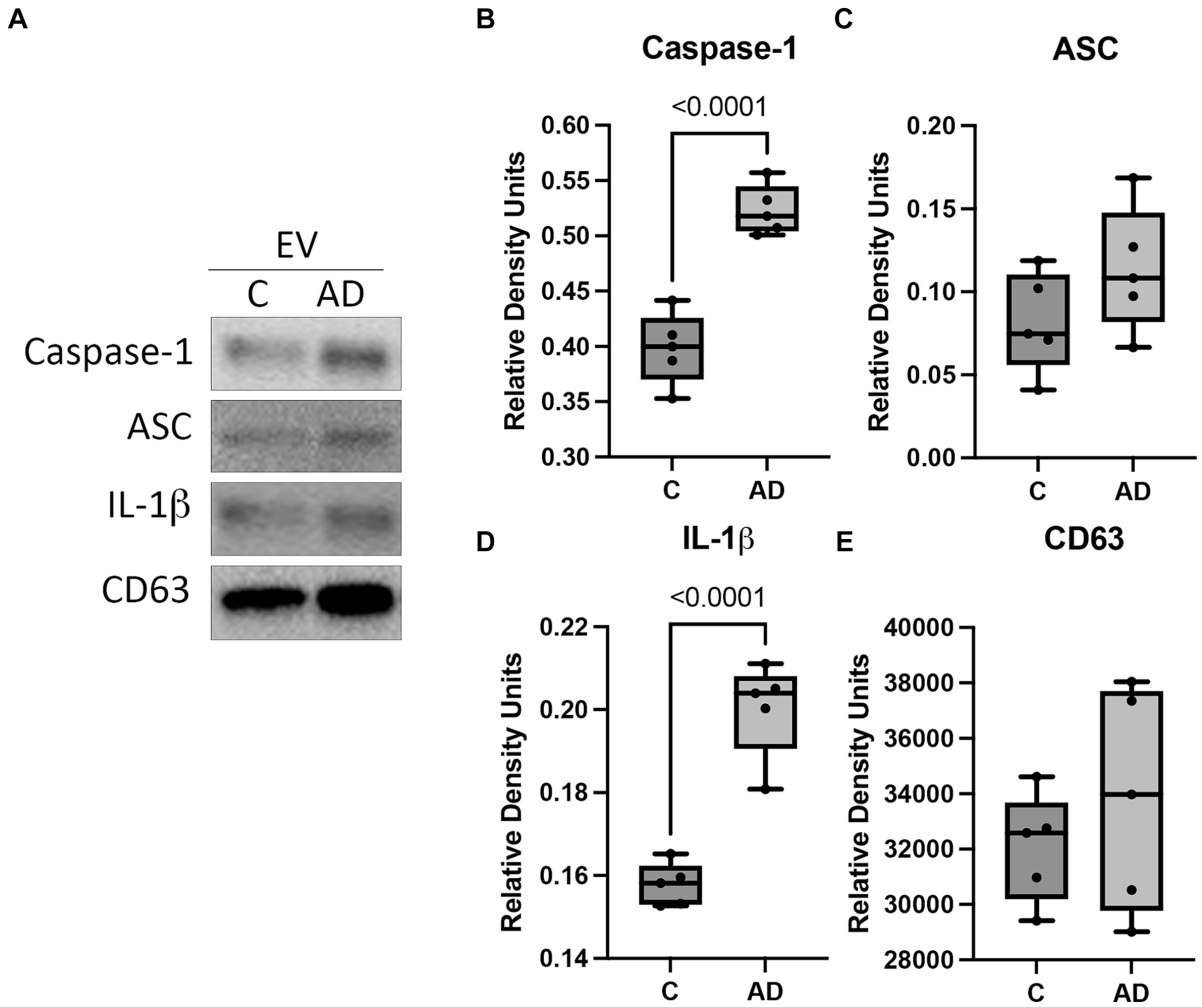
Inflammasome proteins in EV from APP/PS1 mice. Serum-derived EV were isolated from WT control (C) and APP/PS1 mice (AD) and analyzed by **(A)** immunoblot for the expression of **(B)** caspase-1 (active/cleaved), **(C)** ASC, **(D)** IL-1β (active/cleaved), and **(E)** the EV marker CD63. Data presented as box plots with the min to max showing all points. Data normalized to CD63. *N* = 5 per group.

We have previously shown that inflammasome proteins are present in EV after traumatic brain injury and are carried throughout the body inducing an inflammatory response in the lungs (De Rivero Vaccari et al., 2016a; Kerr N. A. et al., 2018; Kerr et al., 2019, 2021) and the heart (Keane et al., 2023). Therefore, we hypothesized that EV may be responsible for inducing an inflammatory response in the heart in AD. Consistent with previous studies, we isolated EV from the serum of AD and control mice and characterized them for number of particles (Supplementary Figure S3A) and particle size (Supplementary Figure S3B), as well as the expression of the inflammasome signaling proteins and the EV marker CD63 (Figure 4A). The protein levels of the inflammasome signaling proteins caspase-1 (Figure 4B) and IL-1β (Figure 4D) were elevated in the serum-derived EV from AD mice when compared to controls, and we found no significant difference in the levels of ASC (Figure 4C) or CD63 (Figure 4E). These results indicate that in AD, there is a heightened level of inflammatory signals circulating the body via EV that contain an inflammasome protein cargo.

### 3.6 Adoptive transfer of AD patient-derived EVs into cardiovascular cells

**Figure 5:**
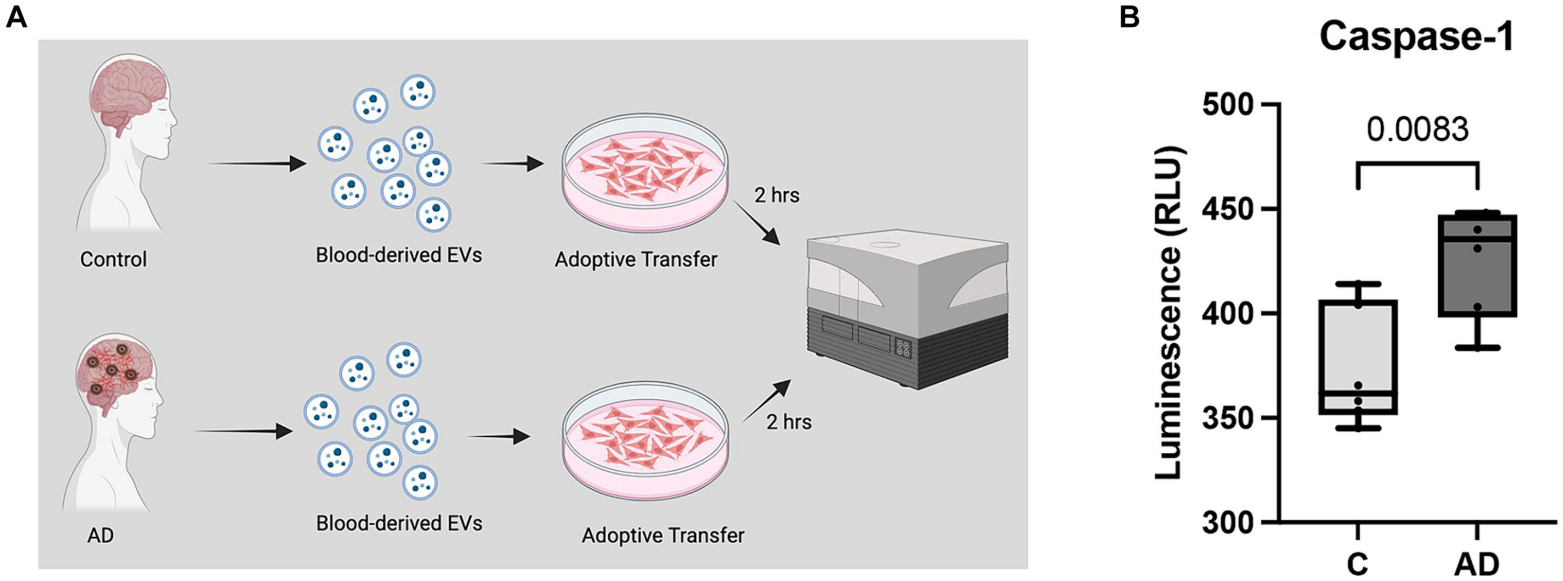
Caspase-1 activity in human cardiovascular cells after EV exposure. **(A)** Caspase-1 activity was measured from cell media collected from cardiovascular cells exposed to EV for 2 h. **(B)** Caspase-1 activity of cell media from cells exposed to serum-derived EV from AD patients and age-matched controls (C). Data presented as box plots with the min to max showing all points. *N* = 6 per group.

**Figure 6:**
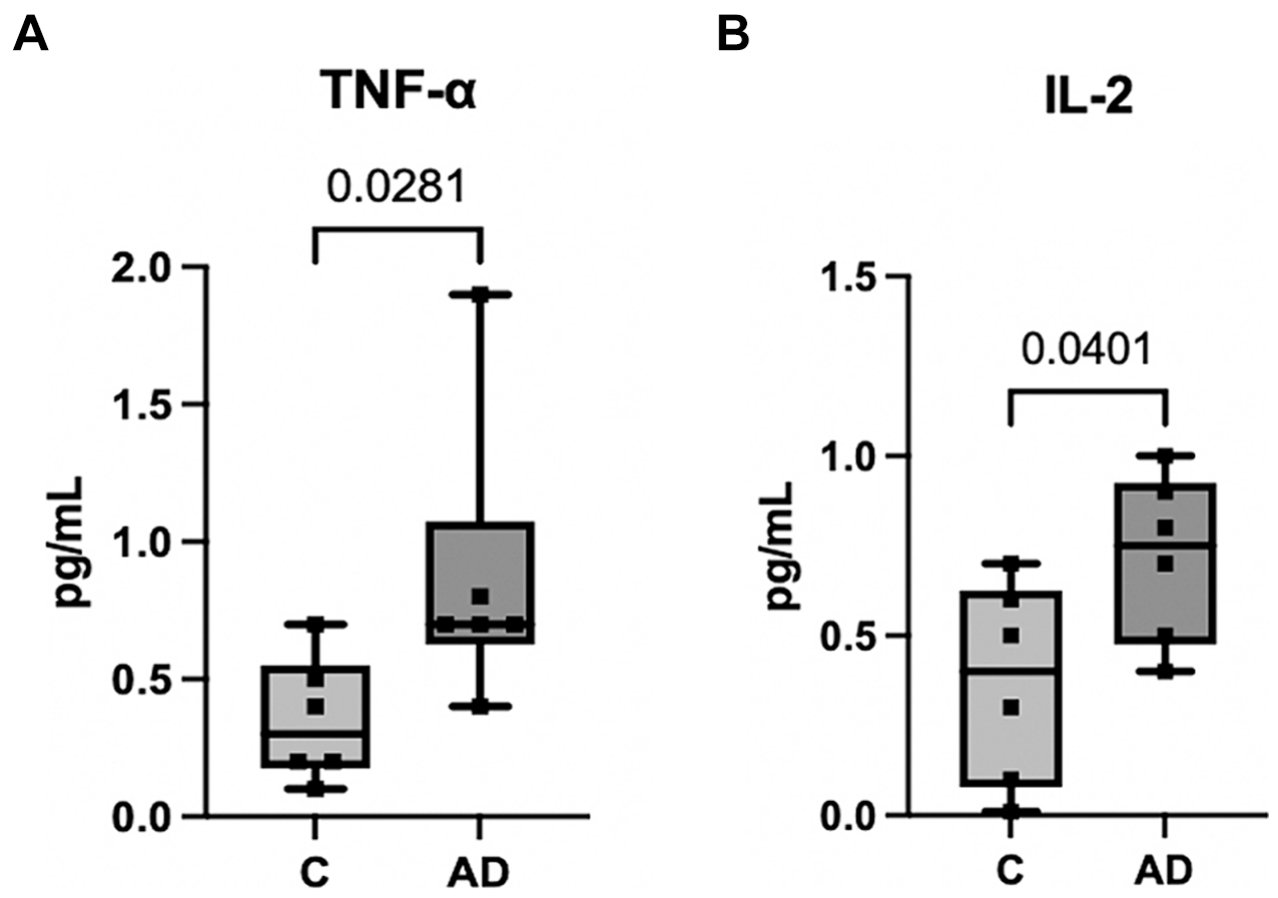
Inflammatory Cytokine activity in human cardiovascular cells after EV exposure. Protein levels in pg./mL of inflammatory cytokines TNF-α **(A)** and IL-2 **(B)** in the cell media from cells exposed to serum-derived EV from AD patients and age-matched controls. Data presented as box plots with the min to max showing all points. *N* = 6 per group.

We have previously shown that a cargo of EV containing inflammasome proteins contributes to comorbidities after TBI (Kerr N. A. et al., 2018; Kerr et al., 2019, 2020; Keane et al., 2023). To determine whether AD-associated EV contribute to inflammasome activation in T/G HA-VSMC human cardiovascular cells, an adoptive transfer experiment of serum-derived EV from AD patients and healthy controls was carried out. Human cardiovascular cells were exposed to isolated EV for 2 h (Figure 5A) and then the cell media was analyzed for the activity of caspase-1 using the Caspase-Glo® 1 Inflammasome Assay. Harvested cell media from cells exposed to AD-derived EV exhibited significantly higher levels of caspase-1 activation than those from cells exposed to EV isolated from age-matched healthy controls (Figure 5B). In addition, we found that the levels of the inflammatory cytokines TNF-α and IL-2 were elevated in the cell media from cells exposed to AD-derived EV when compared to the media of cells exposed to EV isolated from age-matched healthy controls (Figure 6). Moreover, EV from AD patients in media and exposed to no cardiovascular cells, which was used to determine the basal activity of patient-derived EVs, indicated that EV from AD patients when exposed to no cardiovascular cells presented much lower levels of active caspase-1 when compared to the group of cardiovascular cells that were stimulated with EV derived from AD patients (Supplementary Figure S4). Together, our results indicate that EV released from AD patients induce significant inflammation in cardiovascular cells.

## 4 Discussion

In this study, we show that increased levels of inflammasome proteins released during AD are present in the cerebral cortex, serum-derived EV, and the ventricles of the heart. Moreover, Pyrin and NLRP1 inflammasomes are activated in the cerebral cortex of APP/PS1 mice. Additionally, the inflammasome proteins caspase-1 and IL-1β are elevated in serum-derived EV. In cardiac ventricles, the inflammasome proteins caspase-8, ASC, and IL-1β were upregulated; thus, demonstrating activation of a non-canonical inflammasome in the heart of APP/PS1 mice. Adoptive transfer of EV containing inflammasomes proteins from AD patients and age-matched controls into cardiovascular cells resulted in inflammasome activation, suggesting that EV play a role in mediating cardiac inflammation in AD.

AD is a complex disease with many factors contributing to pathology. The accumulation of NFTs and Aβ plaques can activate an inflammatory response in the brain (Ismail et al., 2020). Our results show that two inflammasome sensors, Pyrin and NLRP1, are elevated in the cortex of AD mice, indicating multiple inflammatory triggers governing the inflammatory response associated with AD. Other groups have shown that NLRP1 is upregulated in AD (Tan et al., 2014; Kaushal et al., 2015) and that NLRP1 genetic variants are associated with AD (Pontillo et al., 2012). Furthermore, NLRP1 knockdown was associated with reduced neuronal pyroptosis and improved performance on cognitive tests (Tan et al., 2014). Our recent study has revealed that NLRP1 is primarily expressed in neurons in early stages of AD (Vontell et al., 2023), suggesting that neurons contribute to the innate immune response in the early stages of AD.

Although most AD cases are sporadic, currently there are no well-established rodent models of sporadic AD that mimic the pathological hallmarks of AD. Current efforts to understand familial AD have focused on studying mice that present features that increase the likelihood of developing AD such as ApoE−/− mice, TREM-2 mice or models of vascular risk factors associated with AD (Foidl and Humpel, 2020). In this study, we used a mouse model with a genetic predisposition toward AD in order to better understand the role of EV and heart inflammation in AD mice presenting a pathological hallmark such as Aβ in a well characterized model known to present inflammasome activation. Future studies should consider the effects of different non-genetic contributors to AD pathogenesis as they relate to CNS and peripheral inflammation and the effects of this inflammatory response on AD-related comorbidities.

Our finding that Pyrin is elevated in the cerebral cortex of APP/PS1 mice is novel. The Pyrin inflammasome is activated by homeostasis-altering molecular processes (HAMPs) (Heilig and Broz, 2018), and cytoskeletal changes (Schnappauf et al., 2019). Pyrin plays a role in inflammatory diseases such as Familial Mediterranean Fever (FMF), pyrin-associated autoinflammation with neutrophilic dermatosis (PAAND), and mevalonate kinase deficiency (De Torre-Minguela et al., 2017; Schnappauf et al., 2019; Alehashemi and Goldbach-Mansky, 2020). Both, FMF and PAAND can result in amyloidosis (de Torre-Minguela et al., 2017). The Pyrin inflammasome has also been implicated in mediating the induction of neutrophil adhesion to CNS vasculature (Dumas et al., 2014) and inflammatory responses in the brain after TBI (Keane et al., 2023). Since AD has been associated with extravasation of neutrophils into the brain (Zenaro et al., 2015), it is possible that Pyrin may contribute to AD pathology by increasing amyloid in the brain or by attracting neutrophils to the brain.

Many risk factors of cardiovascular disease are also risk factors for dementia such as hypertension, diabetes, and smoking (Samieri et al., 2018). Cardiovascular comorbidities are common with AD and are associated with poorer outcomes (Zhao et al., 2008). Here, we show that the inflammasome signaling proteins caspase-8, ASC, and IL-1β in the heart ventricles are upregulated in AD mice. Additionally, the oligomerization of ASC into ASC specks was also upregulated in the atria and ventricles of the heart, indicating a role for the inflammasome in heart dysfunction in AD. Even though the monomeric form of ASC was elevated only in the ventricles, we detected increased amounts of ASC specks (oligomerized ASC specks) in the atria and ventricles. This finding indicates that the pathogenic form of ASC interacts with Aβ (Franklin et al., 2014) and that ASC is elevated in both chambers of the heart. This finding is consistent with our previous study in the heart of ASC citrine reporter mice in which ASC specks were elevated on both, the atria and the ventricles after brain injury, even though the monomeric form of ASC was only elevated in one of the chambers (Keane et al., 2023).

Inflammation of the heart leads to chronic heart failure, a diagnosis that usually follows ventricular dysfunction (Dick and Epelman, 2016). Pro-inflammatory cytokines such as IL-1 have been implicated in hemodynamic abnormalities and have toxic effects in the heart (Anker and von Haehling, 2004). The increase in IL-1β and other inflammasome proteins may cause heart failure via inflammation of the ventricles. Although we did not find any significant differences in blood pressure, heart rate, and oximetry to indicate cardiac dysfunction between control and AD mice, there was a trend for these measures to be lower in AD mice than in the control group. This is consistent with previous findings on the accumulation of Aβ and Tau following cerebral hypoperfusion and its effect on AD (Tini et al., 2020). Therefore, future studies are needed to determine whether cardiovascular complications in AD mice may worsen over time. Moreover, in the present study, we used male mice, but we have recently shown that inflammaging in the brain is higher in females than in males. Thus, future studies should consider whether there are sex differences in the inflammatory response present in the heart that is associated with AD pathology (Raval et al., 2019; Cyr and de Rivero Vaccari, 2023b).

NTA was carried out to characterize the EVs for their size and concentration. Our findings indicate that AD mice and WT mice presented the same EV concentration as well as the same particle size. The size is consistent with what we expect to see for EVs with a mean below 150 nm. Moreover, these data indicate that in the APP mouse model, when compared to age-matched WT controls, there is no difference in particle concentration between groups, suggesting that AD pathology does not affect EV release into the bloodstream at 6 months of age.

Our earlier work has shown that inflammasome proteins are present in EV following CNS injury and carry innate immune proteins to peripheral organs such as the lungs (De Rivero Vaccari et al., 2016a; Kerr N. et al., 2018; Kerr N. A. et al., 2018; Kerr et al., 2020) and heart (Keane et al., 2023). Here, we extend this work by examining whether EV are involved in inflammatory signaling between the brain and the heart in AD. Accordingly, the inflammasome signaling proteins caspase-1 and IL-1β are upregulated in serum-derived EV in AD mice. Similarly, there is evidence that EV mediate the spreading of AD pathology within the brain (Vandendriessche et al., 2020; Ruan et al., 2021) and that blood-derived EV are a promising biomarker for AD (Badhwar and Haqqani, 2020; Delgado-Peraza et al., 2021). Currently, it is unknown whether brain inflammation in AD precedes that observed in the heart. However, it is known that cardiovascular problems lead to dementia, particularly in cases of vascular dementia (O'Brien and Thomas, 2015), and that the activation of an immune response can induce heart failure (Dick and Epelman, 2016). Since our earlier work demonstrated that brain-derived EV carry inflammatory signals that result in systemic inflammation (Kerr N. A. et al., 2018; Kerr et al., 2020, 2021), it appears that inflammatory signals in EV are released into blood and alter the inflammatory response in peripheral tissues. Nevertheless, the present work reveals that EV from AD patients, contain a cargo of inflammasome proteins that induce inflammasome activation in cardiovascular cells. Moreover, our findings of elevated levels of TNF-α and IL-2 following adoptive transfer of EV from AD patients when compared to adoptive transfer of EV from healthy controls suggest that in addition to inflammasome activation, the cargo in EV from AD patients is also capable of inducing an inflammatory response that is not directly related to the inflammasome, but the cargo in these EV also activates other important cytokines involved in cardiac inflammation (Rolski and Blyszczuk, 2020; Lagan et al., 2022). However, further studies are needed to determine whether this EV signal travels from the brain to the heart or from the heart to the brain.

We have previously shown that EV carrying inflammasome proteins travel throughout the body reaching the cerebrospinal fluid (CSF) from blood, resulting in exacerbated inflammasome activation (Raval et al., 2019). Similarly, there is a bidirectional communication between the brain and several organs such as the lungs (Kerr N. et al., 2018; Kerr N. A. et al., 2018; Kerr et al., 2020), the heart (Keane et al., 2023) and the gut (Kerr et al., 2022) in which EV carrying inflammasome proteins contribute to systemic inflammation. Moreover, it has been shown that EV carrying inflammasome proteins are capable of traveling throughout the body and cross the BBB to induce brain inflammation (Chavez et al., 2021). In addition, EV of neuronal origin from different cell types are known to be released and contribute to AD pathology (Song et al., 2020; Cai et al., 2022). Together, these data indicate that there is a bidirectional EV communication pathway between the brain and peripheral organs that contribute to inflammation in the CNS and the periphery.

Furthermore, we have shown that EV release blocked with enoxaparin as well as inflammasome inhibition with IC100 decrease inflammasome activation in peripheral organs (Kerr et al., 2021), suggesting that, at least in part, release of EV containing inflammasome proteins contribute to the inflammatory response in the periphery. However, it is likely that other signaling proteins of the immune response beyond the inflammasome that are also released in EV also contribute to the inflammatory response in the periphery.

In conclusion, our data provides evidence that there is a neural-cardiac axis mediated by serum-derived EV in AD. These EV carry inflammasome signaling proteins and induces inflammation in the brain and heart. These findings provide a link between the heart, EV, and the brain. Therefore, the inflammasome may provide a novel therapeutic target for the treatment of cardiac comorbidities in AD and beyond.

## Data availability statement

The raw data supporting the conclusions of this article will be made available by the authors, without undue reservation.

## Ethics statement

The animal study was approved by Animal Care and Use Committee of the University of Miami. The study was conducted in accordance with the local legislation and institutional requirements.

## Author contributions

BC: Data curation, Formal analysis, Investigation, Validation, Visualization, Writing – original draft, Writing – review & editing. EC: Data curation, Formal analysis, Investigation, Methodology, Validation, Visualization, Writing – review & editing. RH: Data curation, Formal analysis, Investigation, Methodology, Project administration, Supervision, Validation, Visualization, Writing – review & editing. WD: Conceptualization, Funding acquisition, Investigation, Methodology, Resources, Writing – review & editing. RK: Data curation, Formal analysis, Funding acquisition, Methodology, Resources, Visualization, Writing – review & editing. JR: Conceptualization, Data curation, Formal analysis, Funding acquisition, Investigation, Methodology, Project administration, Resources, Supervision, Validation, Visualization, Writing – review & editing.
