# Supplementary figures and images for: Extracellular vesicles mediate inflammasome signaling in the brain and heart of Alzheimer’s disease mice
Source: Front Mol Neurosci. 2024 Apr 10;17:1369781. doi: 10.3389/fnmol.2024.1369781 (PMC11039928; doi:10.3389/fnmol.2024.1369781)

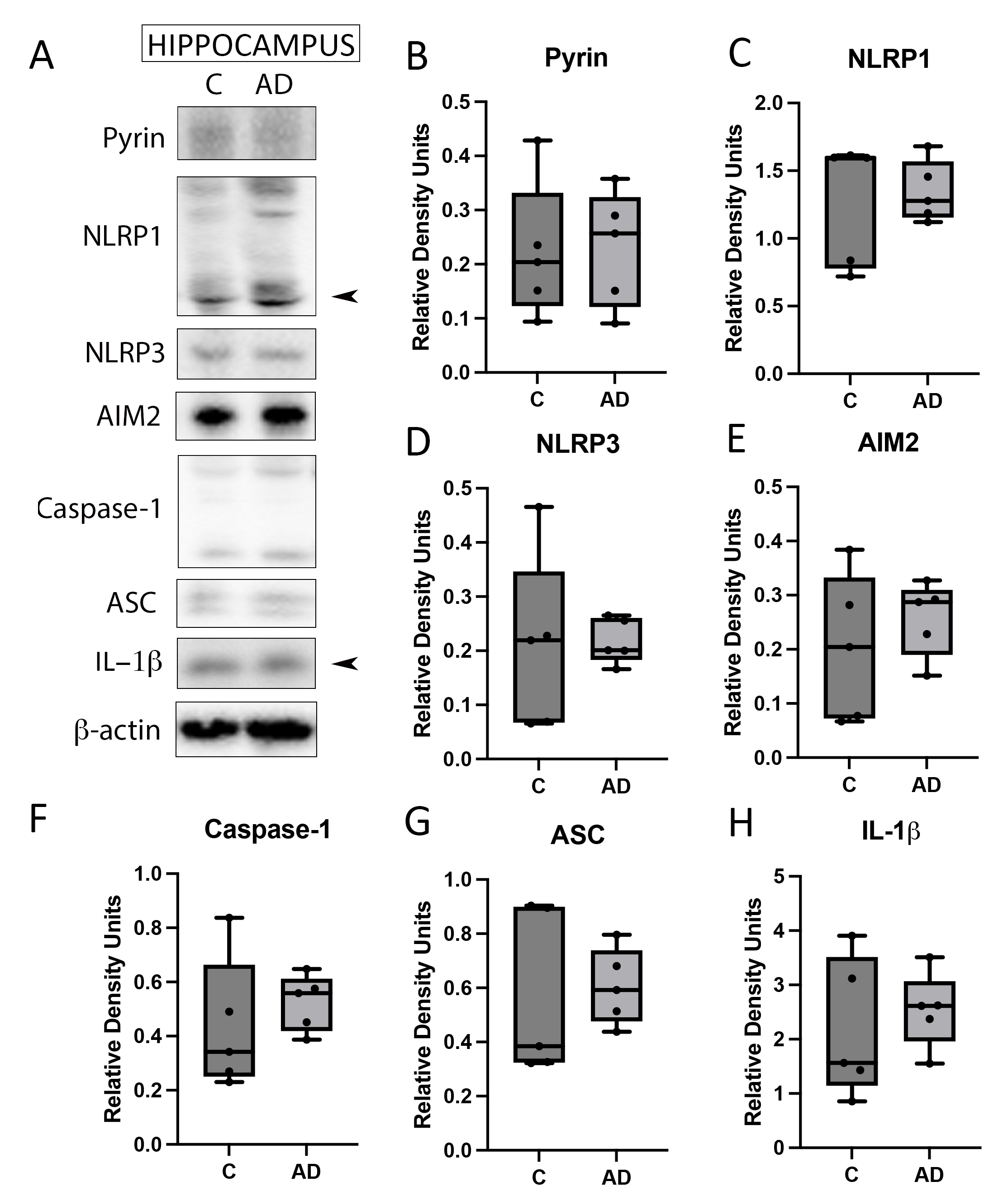

Supplement: Supplementary file 1 [file Image_1.JPEG]

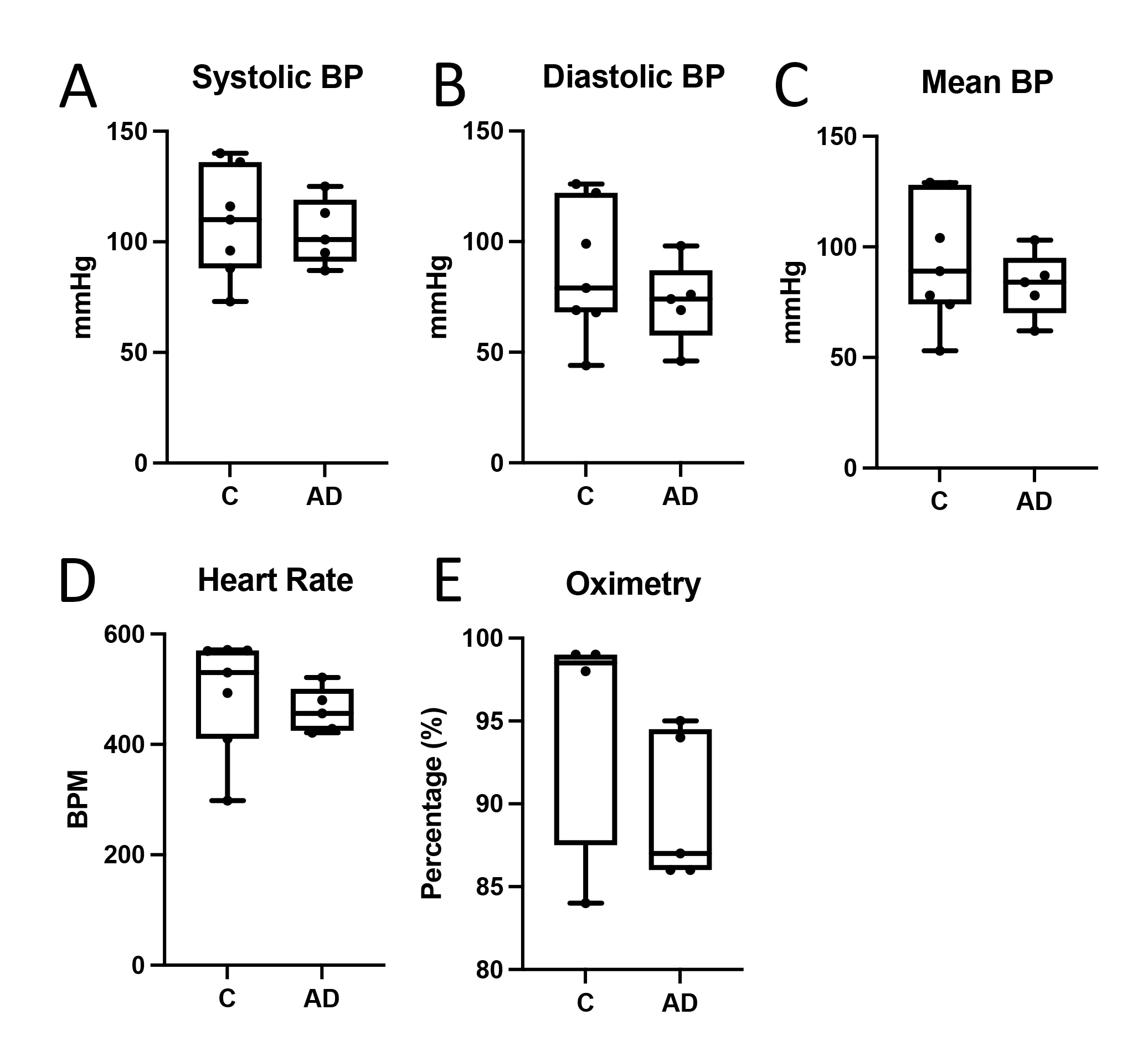

Supplement: Supplementary file 2 [file Image_2.JPEG]

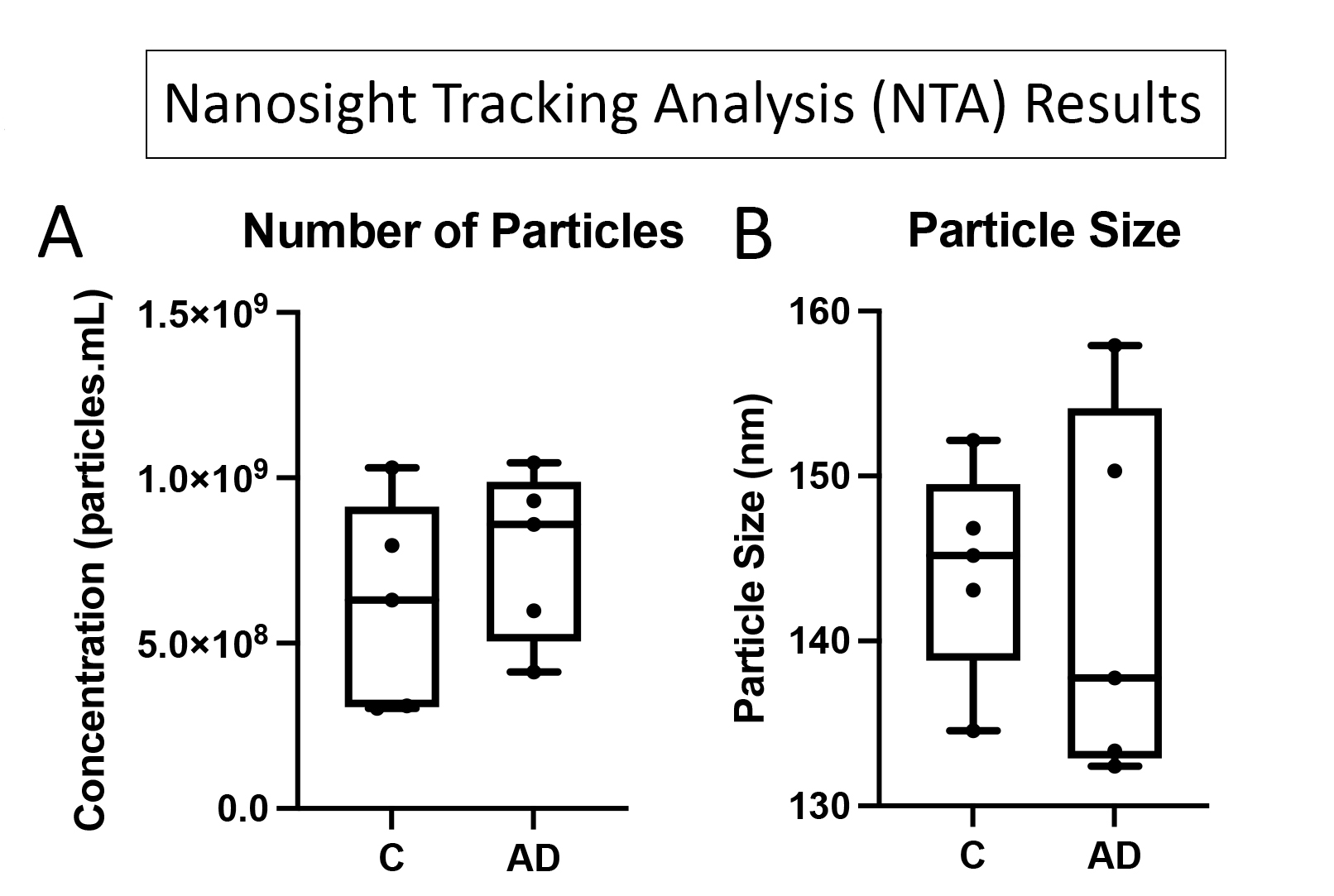

Supplement: Supplementary file 3 [file Image_3.JPEG]

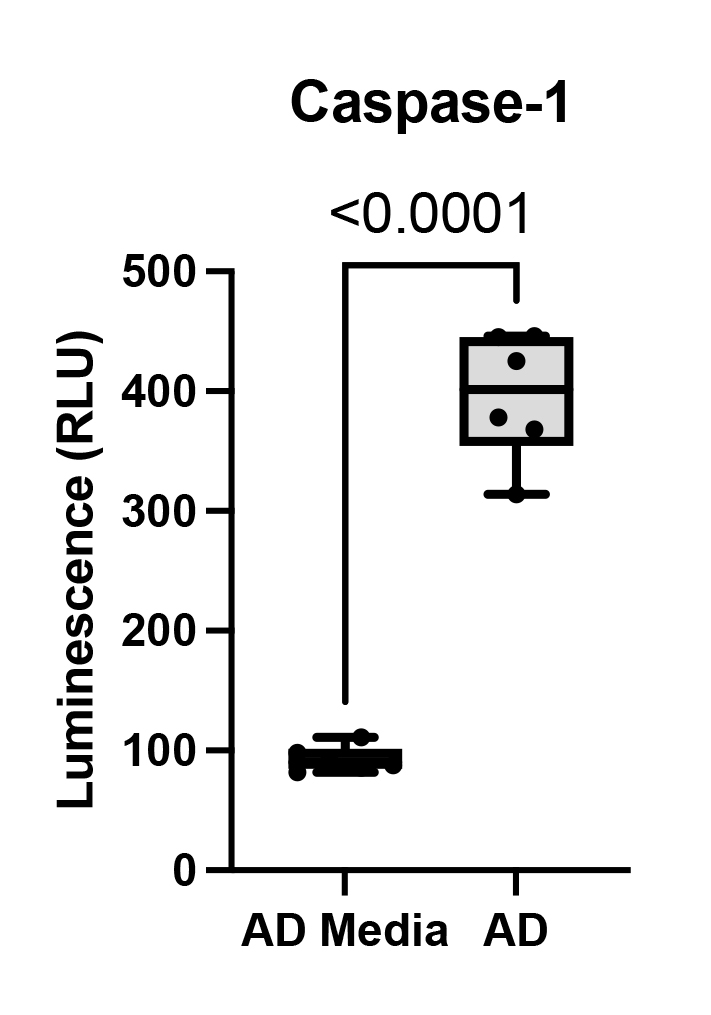

Supplement: Supplementary file 4 [file Image_4.JPEG]
